# CD70 (TNFSF7) is expressed at high prevalence in renal cell carcinomas and is rapidly internalised on antibody binding

**DOI:** 10.1038/sj.bjc.6603222

**Published:** 2006-08-01

**Authors:** P J Adam, J A Terrett, G Steers, L Stockwin, J A Loader, G C Fletcher, L-S Lu, B I Leach, S Mason, A C Stamps, R S Boyd, F Pezzella, K C Gatter, A L Harris

**Affiliations:** 1 Celltech Antibody Centre of Excellence, 216 Bath Road, Slough, Berkshire SL1 4EN, UK; 2 Medarex Inc., 521 Cottonwood Drive, Milpitas, CA 94022, USA; 3 Cancer Research UK Molecular Oncology Laboratories, Weatherall Institute of Molecular Medicine, John Radcliffe Hospital, Oxford OX3 9DS, UK; 4 MRC Toxicology Unit, Hodgkin Building, University of Leicester, P.O. Box 138, Lancaster Rd, Leicester LE1 9HN, UK

**Keywords:** proteomics, renal cell carcinoma (RCC), CD70, antibody, internalisation

## Abstract

In order to identify potential markers of renal cancer, the plasma membrane protein content of renal cell carcinoma (RCC)-derived cell lines was annotated using a proteomics process. One unusual protein identified at high levels in A498 and 786-O cells was CD70 (TNFSF7), a type II transmembrane receptor normally expressed on a subset of B, T and NK cells, where it plays a costimulatory role in immune cell activation. Immunohistochemical analysis of CD70 expression in multiple carcinoma types demonstrated strong CD70 staining in RCC tissues. Metastatic tissues from eight of 11 patients with clear cell RCC were positive for CD70 expression. Immunocytochemical analysis demonstrated that binding of an anti-CD70 antibody to CD70 endogenously expressed on the surface of A498 and 786-O cell lines resulted in the rapid internalisation of the antibody–receptor complex. Coincubation of the internalising anti-CD70 antibody with a saporin-conjugated secondary antibody before addition to A498 cells resulted in 50% cell killing. These data indicate that CD70 represents a potential target antigen for toxin-conjugated therapeutic antibody treatment of RCC.

Advanced renal cell carcinoma (RCC) is generally resistant to chemotherapy treatment (Yagoda, 1995; Motzer, 1999), and immunotherapy with interferon-alpha and interleukin-2 (IL-2) as single agents or in combination with the chemotherapeutic agent 5-fluorouracil is currently used as systemic treatment for some cases. Response rates remain low at around 15%, and the toxicity of high-dose IL-2 limits its use (Krown, 1987; Muss, 1998; Kammula, 1998; van Herpen *et al*, 2000). Targeted therapeutics are relatively new to the oncology field, most acting via the inhibition of mitotic signalling kinases. Therapeutic antibodies have been exploited for their target specificity and low toxicity, and many have already been approved for clinical use, including herceptin (trastuzumab) and erbitux (cetuximab), which target the activities of cell-surface receptors, her2 and EGFR, respectively, in solid tumours. Antibodies can also be used to specifically deliver cytotoxic drugs to tumour cells as immunoconjugates that bind to, and internalise with, highly expressed cell surface antigens. An example of an antigen found to be overexpressed in RCC is carbonic anhydrase IX (CA9), which is the target for the monoclonal antibody (mAb) G250 (Pastorek *et al*, 1994; Uemura *et al*, 1999; Grabmaier *et al*, 2000; Li *et al*, 2001; Bui *et al*, 2003). Immunoscintigraphy of RCC patients dosed with iodine-131-labelled G250 demonstrated tumour targeting to metastatic sites (Divgi *et al*, 1998; Steffens *et al*, 1999; Kennett, 2003).

The aim of this study was to identify other cell-surface proteins specifically associated with RCC that might be suitable targets for antibody-targeted cytotoxic therapeutics. To compile a list of candidate RCC cell-surface proteins, we used a previously described proteomics approach adapted for breast cancer cell lines (Adam *et al*, 2003) using plasma membrane preparations from the A498, SW839 and CAKI-2 RCC-derived cell lines. As described previously (Adam *et al*, 2003), potential targets were analysed further using real-time quantitative RT – PCR and immunohistochemistry to quantify expression in normal tissues and clinical RCC samples. We found CD70, a type II transmembrane cell-surface protein of the tumour necrosis factor (TNF) receptor family (Goodwin *et al*, 1993; Bowman *et al*, 1994; Hintzen *et al*, 1994), to be expressed at high levels in RCC, with expression retained in metastatic RCC tissues. We also show that CD70 is rapidly internalised on binding of an anti-CD70 mAb in RCC-derived cell lines and that this property allows specific killing of the CD70-expressing cells by incubating the primary antibody with a saporin-conjugated secondary antibody. These data demonstrate that CD70 is a selective biomarker for the diagnosis and/or treatment of RCC using cytotoxic immunotherapy.

## MATERIALS AND METHODS

### Preparation of membrane fractions and mass spectrometry

Membrane fractions from the A498 and SW839 RCC-derived cell lines were prepared from 2 × 10^8^ cells as described previously (Adam *et al*, 2003). Purified membrane proteins were resolved on a 20 cm 6% Laemlli one-dimensional gel (BioRad, Hemel Hempstead, UK) and 0.5 mm slices were subjected to trypsinolysis and prepared for MALDI-TOF mass spectrometry (Voyager STR, Applied Biosystems, Framingham, MA, USA) as previously described (Adam *et al*, 2003). Selected peptide masses for CD70 (M+H)=1217.6 and 1142.6 were characterised further by MS/MS using a tandem quadrupole TOF mass spectrometer equipped with a nanospray ion source (Micromass UK Ltd, Manchester, UK). Using the SEQUEST search program (Link *et al*, 1999), ion fragmentation mass spectra of these peptides were sequenced by *γ*- and *β*-ion analysis, and identified by comparison with a FASTA database of public domain proteins constructed of protein entries in the non-redundant database held by the National Center for Biotechnology Information (http://www.ncbi.nlm.nih.gov) and ExPasy (http://www.expasy.com).

### Human tissues and cell lines

A range of normal frozen tissues (brain, breast, kidney, liver, tonsil, lymph nodes, skin and thyroid) and tumour tissues were obtained from the Cellular Pathology Department at the John Radcliffe Hospital, Oxford, UK together with pre-sectioned primary and metastatic RCC tissue from Ardais Corporation, Lexington, MA, USA. Frozen sections of 8 *µ*m were cut for each tissue on a cryostat (Leica Microsystems (UK) Ltd, Milton Keynes, Bucks, UK) and mounted on Snowcoat X-tra glass slides (Surgipath Europe Ltd, Peterborough, UK). Following air-drying for 30 min at room temperature (RT), each section was immersed in 100% acetone for 15 min, air dried and then stored at −20°C until required. The human RCC-derived cell lines SW839 (American Type Culture Collection (ATCC): HTB-49), A498 (ATCC: HTB-44), 786-O (ATCC: CRL-1932) and ACHN (ATCC: CRL-1611) were cultured at 37°C in a humidified atmosphere of 95% air and 5% CO_2_ in growth media specified by the supplier (ATCC, Manassas, VA, USA). A variety of B-cell malignancy derived cell lines (RAMOS, BL16, BL58, AS283, MUTU-III, K562, U937) were kindly provided by Professor Martin JS Dyer, Department of Haematology, University of Leicester, UK. Human PBMCs were purified from donor blood (from the National Blood Service, Bristol, UK) by diluting 1 : 2 in PBS, layering over a 20 ml ficol solution and spinning at 2500 r.p.m. for 20 min.

### Flow cytometry

Harvested cell lines were washed in Dulbecco's Phosphate buffered Salt Solution (DPBS) and resuspended at 1 × 10^7^ cells ml^−1^. Adherent cells were detached by incubation in 0.5 mM EDTA in PBS. Peripheral blood mononuclear cells (PBMCs) were prepared by Ficoll density centrifugation. An aliquot (100 *µ*l) of cell line or PBMCs was then added to each well of a 96-well plate in triplicate. The cells were pelleted by centrifugation and washed once in ice-cold DPBS. DPBS + 5% BSA, containing 1 *µ*g ml^−1^ of anti-CD70 antibody BU69 (Ancell Corporation, Bayport, MN, USA), was then added to each well (200 *µ*l). For control wells, anti-CD70 antibody was substituted for IgG1 isotype control (Serotec Ltd, Oxford, UK). The cells were then incubated on ice for 60 min then washed three times in cold DPBS and resuspended in DPBS + 5%BSA containing 10 *µ*g ml^−1^ of goat anti-mouse Alexa^488^ conjugate (Molecular Probes Inc., Eugene, OR, USA). Cells were then incubated for a further 60 min on ice, followed by three washes in DPBS, where the final wash contained propidium iodide (10 *µ*g ml^−1^). Acquisition of data was performed using a FACScalibur flow cytometer (FL1 + FL3) with an MPM modification and all subsequent data analysis was performed using CellQuest Pro Software (Becton-Dickinson).

### Immunohistochemistry

Tissue sections were first allowed to warm to RT, then immersed for 10 min in 3% hydrogen peroxide in water to quench endogenous peroxidase activity followed by washing in water and then tris-buffered saline (TBS) pH 7.6. A monoclonal mouse anti-human CD70 antibody, HNE.51 (DakoCytomation, Ely, UK), which has been well characterised for CD70 staining on frozen tissues, was applied to the tissues (2 *µ*g ml^−1^ in TBS) for 90 min followed by two 5 min washes in TBS. Secondary antibody from the DakoCytomation Envision anti-mouse system (DakoCytomation, Ely, UK) was applied for 30 min followed by two washes in TBS. Detection was achieved by a 5 min incubation in the presence of 3,3′-diaminobenzidine (DAB+) substrate chromogen, which results in a brown-coloured precipitate at the antigen site. Sections were counter-stained in Gills II haematoxylin (Surgipath Ltd, Richmond, IL, USA) and mounted under glass coverslips using aqueous mounting medium (Faramount, DakoCytomation, Ely, UK).

### Immunocytochemical analysis of antibody internalisation

Cells were seeded at a density of 5 × 10^4^ cells per chamber of an eight-well chamber slide and incubated as normal (37°C, 5% CO_2_, supplier-recommended media) for 24 h. The cells were cooled to 4°C for 20 min to minimise membrane turnover and media were removed and the cells washed carefully in cold DPBS. Then 1 *µ*g ml^−1^ anti-CD70 antibody BU69 (Ancell Corporation, Bayport, MN, USA) and isotype control antibody were prepared in 200 *µ*l cold serum free DMEM/F12 media, added to their respective chambers and incubated at 4°C for 20 min. Cells were washed twice with DPBS and the 0 h samples fixed in 4% paraformaldehyde for 10 min. Warmed media were added to the remaining chambers and the cells incubated at 37°C for 2, 4 and 24 h before fixation. After fixation, the cells were washed twice in DPBS, then blocked/permeabilised for 20 min at RT in 0.1% saponin/5% donkey serum in DPBS. Biotinylated goat anti-mouse IgG diluted 1 : 200 (10 *µ*g ml^−1^) in 0.1% saponin/5% donkey serum/PBS was then added for 1 h at RT followed by three washes in DPBS. Extravidin-Cy3 (Sigma-Aldrich, Poole, UK) diluted 1 : 500 in 0.1% saponin/5% donkey serum/PBS was added for 30 min followed by three washes in PBS. The cells were then mounted in fluorescence enhancing mounting media (DakoCytomation, Ely, UK) and examined using a Leica Microsystems fluorescence microscope with × 63 oil immersion objective.

### Saporin-mediated cell killing assay

Antibodies conjugated to the ribosome-inactivating toxin Saporin (Thorpe *et al*, 1985) were used to evaluate the ability of CD70 to perform as a therapeutic antibody target. Briefly, A498 cells were harvested and resuspended at 1 × 10^5^ cells ml^−1^. An aliquot of cells (200 *µ*l) was added to each well of a 96-well tissue culture plate and grown to confluence. Cells were washed with PBS and labelled on ice with anti-CD70 antibody BU69 (Ancell Corporation, Bayport, MN, USA) or control IgG1 primary (Serotec Ltd, Oxford, UK) at a range of concentrations. After 30 min, labelled cells were washed three times with cold PBS. Saporin-conjugated goat anti-mouse antibody, Hum-Zap (Advanced Targeting Systems, San Diego, CA, USA), or control antibody was then added to each well to a final concentration of 5 *µ*g ml^−1^. After incubation for 24 h, cells and debris were harvested from the plate using trypsin digestion and centrifugation. The recovered cell pellet was then resuspended in 1 ml PBS containing propidium iodide (0.1 *µ*g ml^−1^ final) and percent cell death in each sample calculated by propidium iodide exclusion analysis using a FACScalibur flow cytometer (Becton-Dickinson, Oxford, UK) equipped with Cellquest Pro software.

### Real-time quantitative RT–PCR

Real-time quantitative RT–PCR analysis of gene expression (Morrison *et al*, 1998) was carried out on first-strand cDNA derived from RNA isolated from samples of normal tissues (Clontech, Palo Alto, CA, USA) and RCC tissues (Ardais Corporation, Lexington MA, USA; Peterborough Tissue Bank, Peterborough, UK). All clinical samples were obtained with informed patient consent and ethical approval. Each PCR reaction contained 10 ng first-strand cDNA (prepared from each mRNA sample using Superscript™ reverse transcriptase, Life Technologies, Carlsbad, CA, USA), SYBR green sequence detection reagents (Applied Biosystems, Foster City, CA, USA) and sense and anti-sense primers. All primer pairs traverse at least one intron and test products have been sequenced to confirm specificity before use in these assays. PCR products from all samples were analysed on agarose gels and positives shown to contain a single PCR product of the size predicted from cDNA. No fragments of the size predicted from genomic DNA were detected in any samples, demonstrating the complete absence of genomic DNA contamination. All reactions were run twice and any samples showing a > 10% variation in copy number excluded from the analysis. The CD70 primers used were as follows: F, gctgctttggtcccattggtcg (exon 1); R, gaggtcctgtgtgattcagctg (exon 2/3 junction; 141 bp product). The CA9 primers used were as follows: F, cagtgcctatgagcagttgctg (exon 6); R, cttagcactcagcatcactgtc (exon 7; 204 bp product). The PCR conditions used for both sets of primers were one cycle at 50°C for 2 min, one cycle at 95°C for 10 min, and 40 cycles of 95°C for 15 s, 65°C for 1 min. Reaction products were assayed on an ABI Prism 7700 Sequence Detection System (Applied Biosystems, Foster City, CA, USA) and the accumulation of PCR product was measured in real time as the increase in SYBR green fluorescence. Data were analysed using the Sequence Detector program v1.6.3 (Applied Biosystems, Foster City, CA, USA). Standard curves relating initial template copy number to fluorescence and amplification cycle were generated using the amplified PCR product as a template, and were used to calculate copy number in each sample. Data were expressed as copy number per nanogram cDNA.

## RESULTS

### Proteomic discovery of CD70 in RCC-derived cell lines

**Figure 1:**
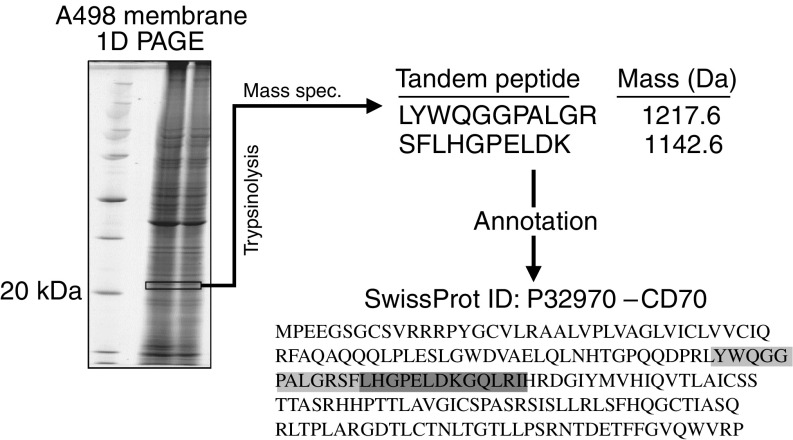
Proteomic discovery of CD70 in the plasma membrane fraction of A498 and SW839 renal carcinoma-derived cell lines. The plasma membrane fraction of A498 and SW839 cell lines was resolved by one-dimensional PAGE. The proteins contained within 0.5 mm consecutive slices of the gel were subjected to trypsinolysis and analysed by MALDI-TOF mass spectrometry with selected masses characterised further by MS/MS using a quadrupole TOF mass spectrometer equipped with a nanospray ion spray. Using the SEQUEST search program, two uninterpreted ion fragmentation mass spectra representing peptides LYWQGGPALGR and SFLHGPELDK identified from the tryptic peptides isolated from a gel slice taken at around 23 kDa were searched against a FASTA database of public domain proteins and were found to match CD70 (SwissProt accession P32970).

Purified cell membrane protein preparations were isolated from the A498, SW839 and CAKI-2 RCC-derived cell lines and individually separated by one-dimensional PAGE. Sequential 0.5 mm gel slices containing the proteins were subjected to trypsinolysis, and the resulting peptide fragments analysed by MALDI-TOF and MS/MS (Figure 1). Two ion fragmentation mass spectra from two tryptic peptides of masses 1217.6 and 1142.6 Da, respectively, were identified in gel slices corresponding to a molecular weight 23 kDa from the A498 and SW839 RCC-derived cell lines (Figure 1). Using the SEQUEST algorithm (Link *et al*, 1999), interpretations of these spectra were searched against a FASTA database of public domain proteins and found to uniquely match two peptide sequences (LYWQGGPALGR and SFLHGPELDK) that unambiguously identify the CD70 antigen (Swiss-Prot accession P32970), a type II transmembrane receptor also known as CD27L or TNF ligand superfamily member 7 (TNFSF7) (Figure 1).

**Figure 2:**
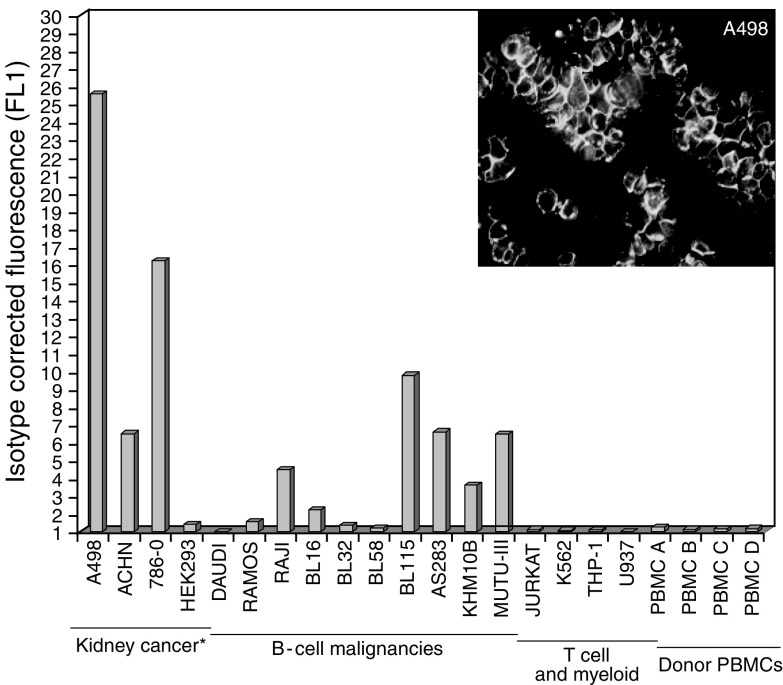
CD70 protein is expressed at the cell-surface of kidney cancer-derived cell lines. An anti-CD70 mAb was used in the context of FACS analysis to determine relative cell-surface protein levels for A498, 786-0, SW839 and ACHN renal carcinoma cell lines. The immortalised embryonic kidney line HEK293 was included as a ‘normal’ kidney cell line control. In addition, as CD70 expression was first described in B/T cells, a panel of cell lines with this derivation was included. The CD19+ or CD3+ subsets of PBMCs were also analysed to confirm the previously observed absence of binding to these cells. Results demonstrated that expression of CD70 was highest in A498 and 786-0 cells. Immunocytochemical analysis was subsequently used to support the finding that CD70 antibody binds to the surface of A498 cells (inset).

To confirm and determine the relative cell-surface expression levels of CD70 in RCC-derived cell lines, the anti-CD70 mAb BU69 was used in FACS analysis. This antibody was selected for FACS and immunocytochemistry as it gave more consistent results in these analyses. The A498, 786-0, and ACHN RCC cell lines and the immortalised HEK293 ‘normal’ kidney cell line were included for FACS analysis, and, as CD70 expression was first described in activated B and T cells, a panel of cell lines with this derivation was also included in the analysis as were the CD19+ and CD3+ subsets of PBMCs. Results demonstrated that expression of CD70 was highest in the A498, then 786-0 RCC-derived cells (Figure 2). Expression in the ACHN cell line was approximately five-fold lower than on the A498 cell line. Cell-surface expression was also detected on the Raji, BL115, AS283, KHM10B and Mutu-III cell lines that are all derived from B-cell malignancies; however, expression on these cell lines was 2–3-fold less than the A498 and 786-O cells (Figure 2). There was no detectable CD70 expression in the T and myeloid cell lines or donor PBMCs analysed. Immunocytochemical analysis with BU69 antibody demonstrated strong membrane reactivity in A498 cells (Figure 2). Scatchard analysis showed that A498, 786-O and ACHN cell lines endogenously expressed 342 000, 265 000 and 33 000 receptors per cell, respectively. FACS and mRNA analyses were also used to determine whether the RCC cell lines expressing CD70 also expressed CD27, the costimulatory receptor for CD70. No detectable CD27 expression could be seen on any of the RCC cell lines tested (data not shown).

### CD70 is expressed in primary and metastatic RCC tissues

**Figure 3:**
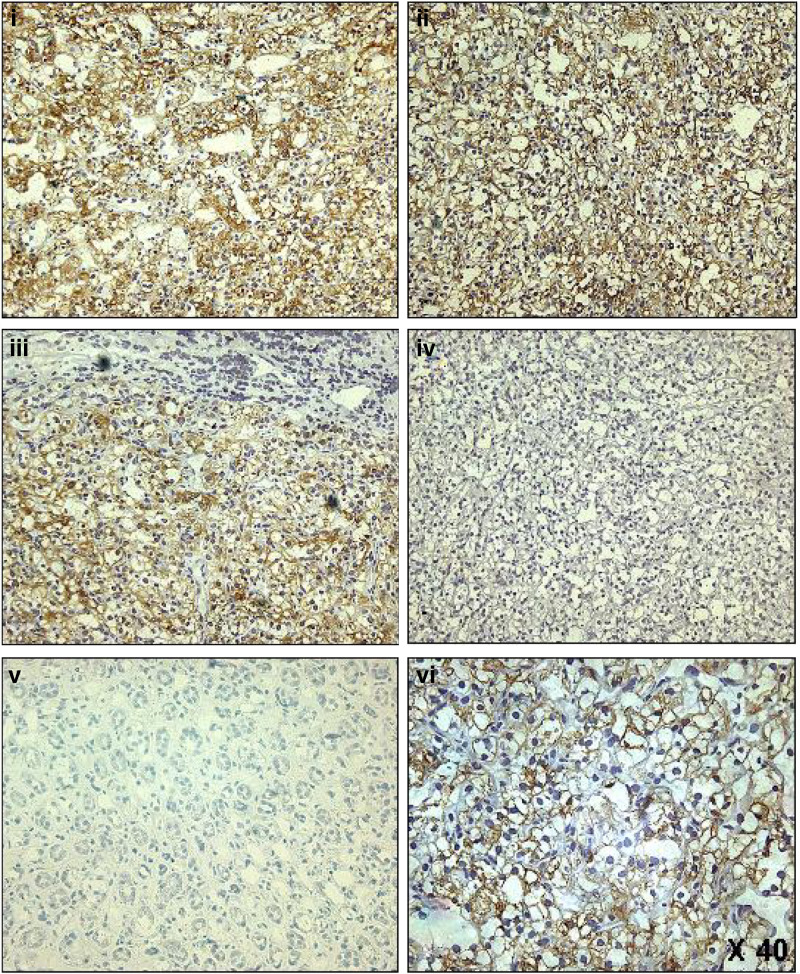
Immunohistochemical analysis of CD70 in RCCs. CD70 expression throughout the carcinoma tissues of frozen sections of clear cell RCC (ccRCC) is shown (panels i–iii) relative to an isotype matched control antibody (panel iv). CD70 expression was not seen in normal kidney tissues (panel v). Panels i–v all show images at × 20 magnification. Panel vi shows a higher power (× 40) image of a CD70-positive ccRCC tissue demonstrating the plasma membrane expression of CD70 in the carcinoma cells.

Having discovered high levels of cell-surface CD70 expression on a number of RCC-derived cell lines, we sought to investigate CD70 expression in clinical RCC tissues. Immunohistochemistry on frozen tissue sections was used to determine expression of CD70 on a number of different types of malignant tissues including 30 kidney cancer donor tissues. The results of this analysis are shown in Table 1. In total, 90 donor tumour tissues were examined; however, only RCC tissues showed strong staining with the anti-CD70 antibody, with 16 out of 30 (53%) cases showing CD70 immunoreactivity. Of all the other tumour tissues examined, weak staining was observed in two of four cases of lymphoblastic lymphoma, four of six cases of large cell lymphoma and one of nine cases of lung adenocarcinoma (Table 1). Examples of CD70 immunostaining of RCC tissues but not normal kidney are shown in Figure 3.

**Figure 4:**
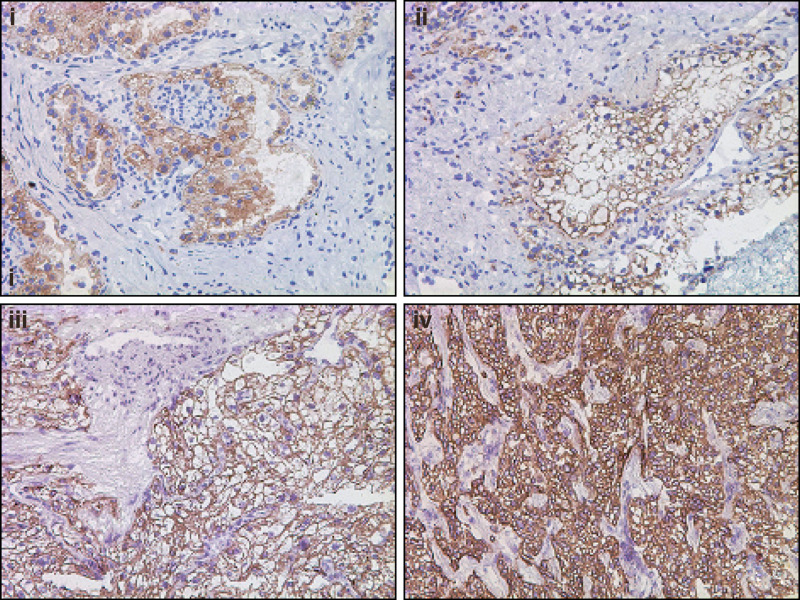
Immunohistochemical analysis demonstrating that CD70 expression is retained in metastatic tissues derived from primary ccRCC donors. CD70 expression in a primary ccRCC tissue (panel i) is shown alongside expression in a section of adrenal gland metastasis from the same donor (panel ii). Panels iii and iv show CD70 expression in retroperitoneal region metastasis and lung metastasis respectively from two separate donors with primary ccRCC. Magnification × 20.

Expression of CD70 was also examined in 11 clear cell RCC (ccRCC) metastatic patient tissues, two of which had matched primary ccRCC tumour tissue for comparison. Eight out of eleven (73%) metastatic tissues showed some CD70 expression, and for the two patients with matched primary and metastatic tissues the intensity of CD70 immunostaining of the primary ccRCC and metastasis was in each case the same. Examples of CD70 immunostaining of metastatic ccRCC tissues are shown in Figure 4, in which patient matched primary and metastatic ccRCC CD70 immunostaining is shown (Figure 4, i and ii).

### CD70 is rapidly internalised on antibody binding to RCC-derived cell lines

**Figure 5:**
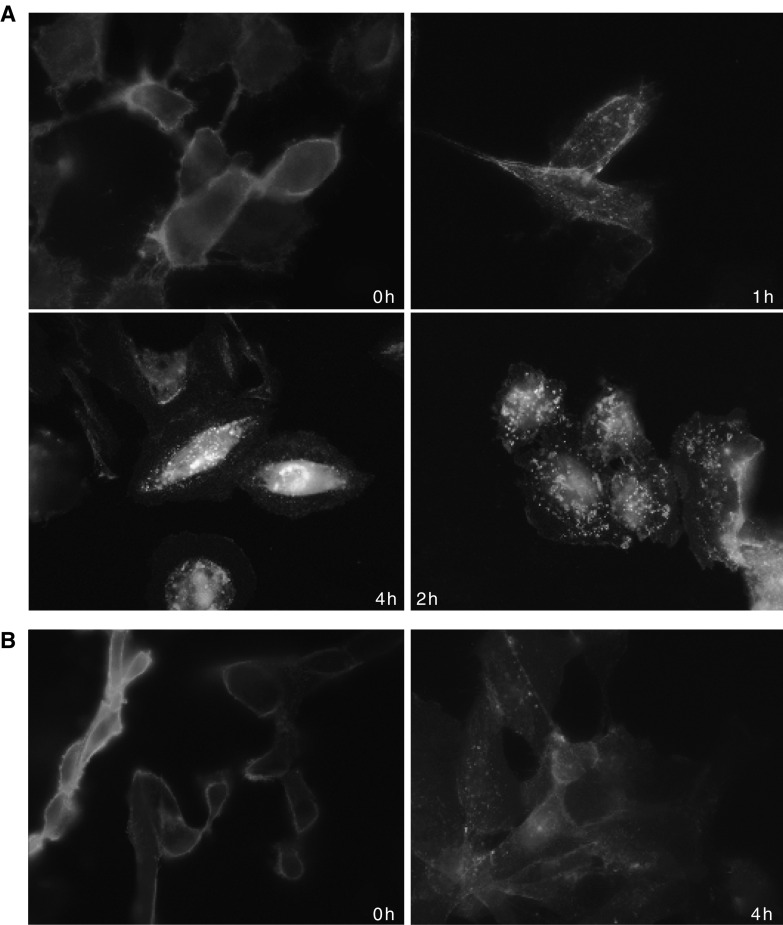
Immunocytochemical analysis demonstrating rapid CD70 internalisation on anti-CD70 antibody binding. (**A**) A498 cells showing anti-CD70 antibody binding to the plasma membrane after incubation at 4°C for 20 min (0 h) followed by clear evidence of internalisation of the antibody (endosomal compartmentalisation) at 1, 2 and 4 h after the addition of growth media at 37°C. (**B**) 786-O cells showing anti-CD70 antibody membrane staining of the cells at 0 h and internalisation after 4 h incubation.

To determine whether CD70 is a suitable target for cytotoxic-conjugated antibody therapy, we investigated CD70 internalisation on antibody binding. An anti-CD70 antibody, BU69, which is suitable for flow cytometric applications, was added to live A498 and 786-O cells that had been cooled to minimise membrane turnover. After a 20 min incubation at 4°C, a proportion of the cells were fixed to represent a 0 h time point, and warmed serum was added to the remainder of the cells that were subsequently fixed at 1, 2 and 4 h time points. Evidence of internalisation of the anti-CD70 antibody–receptor complex was visualised immunocytochemically as described in Materials and methods (Figure 5). Results showed that for both A498 and 786-O cells at 0 h, there was clear plasma membrane staining only with the anti-CD70 antibody. However, after 1 h of incubation, there was clear internalisation of the antibody–receptor complex as evidenced by a reduction in the intensity of plasma membrane staining and appearance of antibody-containing vesicles within the cells (Figure 5). Internalisation was even more pronounced at 2 h and after 4 h the antibody had completely internalised.

**Figure 6:**
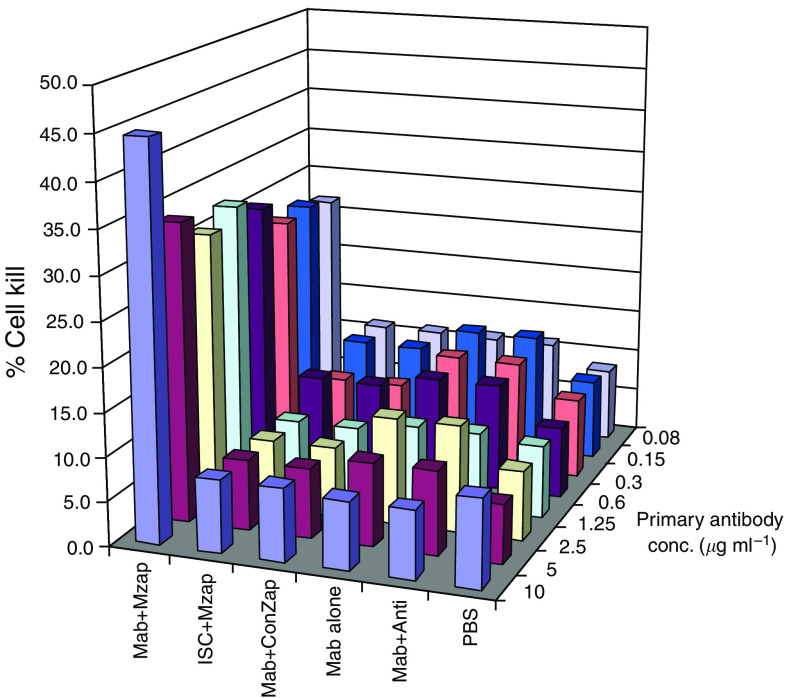
Targeting CD70 on A498 cells with an anti-CD70 mAb and Saporin-conjugated secondary antibody results in specific cell kill. Histograms show the % cell kill following incubation in a range of primary antibody concentrations. High levels of cell death were observed only for cells incubated with anti-CD70 and Saporin-conjugated secondary antibody. Propidium iodide exclusion analysis was used to calculate the % cell kill. Mab=anti-CD70, Mzap=goat anti-mouse Saporin conjugate, ISC=IgG1 isotype control, ConZap=Saporin-conjugated goat IgG, anti=goat anti-mouse IgG. Data shown are representative of three separate experiments.

To investigate the ability of the anti-CD70 antibody–receptor complex to internalise with a toxin and kill RCC cell lines endogenously expressing CD70, we coincubated the anti-CD70 antibody with an anti-mouse secondary antibody that was conjugated to saporin before addition to the A498 cell line for 24 h. This demonstrated that using 10 *µ*g ml^−1^ primary antibody in the presence of 5 *µ*g ml^−1^ saporin-conjugated secondary antibody, approximately 45% of the cells were killed by the addition of anti-CD70 antibody (Figure 6). In contrast, all the appropriate control incubations showed approximately 5% cell death. A range of other primary antibody concentrations was used, with significant cell killing (25%) observed with 0.08 *µ*g ml^−1^, the lowest concentration of primary antibody tested (Figure 6).

### CD70 exhibits a more restricted normal tissue distribution and higher prevalence of expression in RCC tissues than CA9

**Figure 7:**
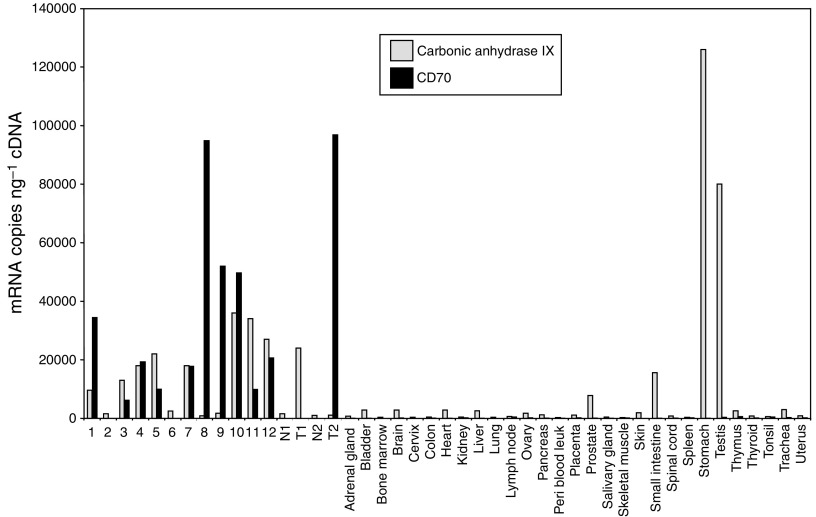
Comparison of the mRNA expression levels of CD70 and CA9 in normal and RCC tissues. Real-time quantitative RT–PCR analysis was used to calculate the mRNA copies per ng cDNA of CD70 and CA9 in a range of 29 normal tissues and 14 renal cancers: 1=renal cell carcinoma containing both granular and clear cells; 2=chromophobe adenocarcinoma; 3=RCC having a tubular pattern with slightly oxyphilic cells; 4=unknown kidney cancer; 5=transitional cell carcinoma arising from renal pelvis; 6=unknown kidney cancer; 7=renal cell carcinoma of clear cell type; 8=Wilms tumour; 9–12=clear cell renal carcinoma; two RCC donors with matched adjacent normal tissue (N=normal, T=tumour).

CA9 is a known antigen currently being targeted for RCC immunotherapy (Divgi *et al*, 1998; Steffens *et al*, 1999; Uemura *et al*, 1999; Grabmaier *et al*, 2000; Li *et al*, 2001; Bui *et al*, 2003; Kennett, 2003). To compare expression of CD70 with CA9, we performed real-time quantitative RT–PCR analysis on multiple normal tissues and clinical RCC samples (Figure 7). These data showed that there was no detectable CD70 mRNA in any of the 29 normal tissues examined; in the thymus, reported expression of CD70 in the medullary epithelium (Hintzen *et al*, 1994; Tesselaar *et al*, 2003) was likely diluted below the limit of detection by the presence in this tissue of other, non-expressing cell types. In contrast, there were high levels of CA9 in the stomach and testis, and to a lesser extent in the small intestine and prostate. In the clinical RCC samples, the overall level and prevalence of CD70 mRNA was greater than that of CA9 (Figure 7).

## DISCUSSION

Identification of antigens expressed specifically and at high levels on kidney cancer cells is a key step in the development of effective immunotherapies for this poorly treated disease. In this paper, we describe the identification of CD70 using proteomic analysis of the plasma membrane fraction of RCC-derived cell lines. CD70 has a very restricted normal tissue expression but was found to be expressed on 16 of 30 RCC clinical specimens and expression was retained in metastatic tissues from ccRCC. In addition, we have demonstrated that binding of an anti-CD70 antibody to cell lines endogenously expressing CD70 results in the rapid internalisation of the antibody–receptor complex, and can mediate cell killing via a toxic immunoconjugate.

CD70 is a type II transmembrane protein that was originally identified as a member of the TNF receptor superfamily with an expression pattern in normal tissues restricted to germinal centre B cells, stromal cells of the thymic medulla and scattered T cells (Goodwin *et al*, 1993; Bowman *et al*, 1994; Hintzen *et al*, 1994). Subsequent studies have described overexpression of CD70 protein in mantle cell lymphoma samples (Zhu *et al*, 2002); on chronic lymphocytic leukaemia B cells (Lens *et al*, 1995; Ranheim *et al*, 1995); and in the tumour cells of thymic carcinomas (Hishima *et al*, 2000), undifferentiated nasopharyngeal carcinomas (Agathanggelou *et al*, 1995), gliomas and meningiomas (Held-Feindt and Mentlein, 2002). Additionally, CD70 overexpression was detected in a multidrug-resistant colonic cell line (SW620-MDR) (Fan *et al*, 2004), and as an inducible gene in TNF-alpha-stimulated human bronchial–epithelial cell line BEAS-2B (Wolf *et al*, 2002), and irradiated glioma cell lines (Wischhusen *et al*, 2002). Our study also identified CD70 expression on B-cell malignancy-derived cell lines using FACS analysis (Figure 2), and on six out of twenty clinical lymphoma specimens by immunostaining (Table 1). Anti-CD70 immunostaining on positive RCC sections was much stronger than that of the lymphomas in our study, and quantitative RT–PCR results also showed an overall higher level of CD70 gene transcription in RCC, indicating that the protein is highly over-expressed.

CD70 is the ligand for CD27; studies of this interaction have shown that CD70 promotes cell survival and expansion of antigen-primed CD8+ T cells, formation of memory T cells and proliferation of B cells (Brown *et al*, 1995; Hintzen *et al*, 1995; Borst *et al*, 2005). CD70 protein, whether expressed on dendritic cells (Hintzen *et al*, 1995), B cells (Rowley and Al-Shamkhani, 2004), tumour cells (Arens *et al*, 2001), or introduced as soluble protein (Kelly *et al*, 2002), promotes the survival and expansion of antigen-primed CD8+ T cells in the mouse, through dose-dependent stimulation of CD27 activity. CD70–CD27 interactions may also have a role in memory T-cell formation, which is reduced by CD70 blockade and enhanced by CD70 overexpression or recombinant protein infusion (Borst *et al*, 2005). The effect of CD70 on B cells is less understood, although *in vitro* studies suggest that the CD70–CD27 interaction promotes plasma cell differentiation (Cormary *et al*, 2004), whereas other experiments have demonstrated CD70-driven B-cell proliferation and immunoglobulin synthesis (Agematsu *et al*, 1995; Kobata *et al*, 1995). Effects in humans may be more significant than in mice owing to higher and more consistent expression of CD27 in human B cells.

Our studies showed a high-level expression of CD70 on RCC cells that might be expected to induce a strong immune response against the tumour. Frequent expression of CD70 on RCC was recently also reported by Junker *et al* (2005). Although RCC is known to be a highly immunogenic tumour, it is also a very aggressive cancer that is clearly able to escape immune surveillance. Many other factors are involved in the generation of an immune response and it has already been shown that CD70-driven CD8+ T-cell expansion is dependent upon priming. The high expression of CD70 observed in RCC may be owing to a partial T-cell induction response, or even simply an incidental result of activation/amplification of a genomic locus.

We also demonstrated that CD70 on RCC cell lines internalises rapidly on specific antibody binding, and that this internalisation can be utilised to transport active cytotoxic molecules into cancer cells. This was corroborated by a recent paper describing an anti-CD70 immunoconjugate of doxorubicin that displayed antibody-mediated toxicity against RCC cell lines (Jeffrey *et al*, 2006).

The development of effective toxin-conjugated immunotherapies will require target antigens that exhibit a highly restricted expression profile in normal tissues, high expression in primary and metastatic cancer tissues and rapid internalisation of the antigen–antibody–toxin complex. CD70 is an antigen that fulfils all of these criteria, in that it is highly overexpressed in a large proportion of renal carcinomas, whereas normal tissue expression is low and restricted to a subset of peripheral blood lymphocytes, and internalises in response to specific antibody binding.

## Figures and Tables

**Table 1:** List of frozen human malignant tissues immunostained with a monoclonal anti-CD70 antibody

**Tumour type**	**No. of cases**	**Positivity**
*Bladder*		
Transitional cell carcinoma	2	—
*Brain*	1	—
*Breast*		
Primary invasive ductal carcinoma	11	—
*Breast node*		
Invasive carcinoma	2	—

*Colon*		
Adenocarcinoma	2	—

*Kidney*		
Renal papillary carcinoma	3	—
Clear cell carcinoma	20	16
Oncocytoma	1	—
Sarcomatoid	1	—
Transitional cell carcinoma	1	—
Neuroblastoma	1	—
Chromophobe	1	—
Wilms tumour	3	—

*Liver*		
Hepatoblastoma	1	—

*Lymphoma*		
Follicular	8	—
Lymphoblastic	4	2
Large cell lymphoma	6	4
B-chronic lymphoid leukaemia	2	—

*Skin*		
Melanoma	1	—

*Thyroid*		
Adenocarcinoma	1	—

*Lung*		
Adenocarcinoma	9	1
Squamous carcinoma	8	—
Clear cell carcinoma	1	—

The number of cases tested for each tumour type is shown along with the number that were CD70 positive (—denotes that none of the tissues tested were CD70 positive).
